# Addressing Missing Data Challenges in Geriatric Health Monitoring: A Study of Statistical and Machine Learning Imputation Methods

**DOI:** 10.3390/s25030614

**Published:** 2025-01-21

**Authors:** Gabriel-Vasilică Sasu, Bogdan-Iulian Ciubotaru, Nicolae Goga, Andrei Vasilățeanu

**Affiliations:** 1 Faculty of Automatic Control and Computers, National University of Science and Technology Politehnica Bucharest, 060042 Bucharest, Romania; sasugabriel94@gmail.com; 2 Military Equipment and Technologies Research Agency (METRA), Ministry of National Defence, Clinceni, 077025 Ilfov, Romania; ciubotarubogdaniulian@gmail.com; 3 The Faculty of Engineering in Foreign Languages, National University of Science and Technology Politehnica Bucharest, 060042 Bucharest, Romania; n.goga@rug.nl

**Keywords:** missing data imputation, geriatric healthcare, frailty detection, MCAR, MAR, MNAR, machine learning, deep learning, wearable sensors, health monitoring systems

## Abstract

In geriatric healthcare, missing data pose significant challenges, especially in systems used for frailty monitoring in elderly individuals. This study explores advanced imputation techniques used to enhance data quality and maintain model performance in a system designed to detect frailty insights. We introduce missing data mechanisms—Missing Completely at Random (MCAR), Missing at Random (MAR), and Missing Not at Random (MNAR)—into a dataset collected from smart bracelets, simulating real-world conditions. Imputation methods, including Expectation–Maximization (EM), matrix completion, Bayesian networks, K-Nearest Neighbors (KNN), Support Vector Machines (SVMs), Generative Adversarial Imputation Networks (GAINs), Variational Autoencoder (VAE), and GRU-D, were evaluated based on normalized Mean Squared Error (MSE), Mean Absolute Error (MAE), and R^2^ metrics. The results demonstrate that KNN and SVM consistently outperform other methods across all three mechanisms due to their ability to adapt to diverse patterns of missingness. Specifically, KNN and SVM excel in MAR conditions by leveraging observed data relationships to accurately infer missing values, while their robustness to randomness enables superior performance under MCAR scenarios. In MNAR contexts, KNN and SVM effectively handle unobserved dependencies by identifying underlying patterns in the data, outperforming methods like GRU-D and VAE. These findings highlight the importance of selecting imputation methods based on the characteristics of missing data mechanisms, emphasizing the versatility and reliability of KNN and SVM in healthcare applications. This study advocates for hybrid approaches in healthcare applications like the cINnAMON project, which supports elderly individuals at risk of frailty through non-intrusive home monitoring systems.

## 1. Introduction

In the data analysis and artificial intelligence domains, missing data present a significant challenge, particularly in fields where accurate and complete datasets are critical for decision-making. One such field is geriatric healthcare, where comprehensive data are essential for the early detection and management of frailty—a syndrome that affects the elderly and impacts their overall quality of life and health outcomes. In our recent study on the Frailty Insights Detection System (FIDS) [1], we developed a comprehensive and intuitive dashboard using artificial intelligence and web technologies to monitor frailty indicators in elderly individuals. This system relies on various data collected through non-intrusive methods to provide healthcare professionals with real-time insights related to individuals’ health status. Missing data represent a critical concern, as they can affect the reliability and effectiveness of such systems.

Missing data can arise due to various reasons, including device malfunctions, user non-compliance, or environmental factors, leading to incomplete records that may distort analysis and predictions. Understanding and addressing missing data are crucial for improving the robustness of health monitoring systems as well as enhancing the overall accuracy of frailty insights detection. In this article, we will explore the types and sources of missing data, the implications of missing data on healthcare analytics, and the methods used to handle and mitigate their impact, particularly in the context of systems designed to monitor and manage frailty in elderly populations.

Missing data are commonly classified into three categories: Missing Completely at Random (MCAR), Missing at Random (MAR), and Missing Not at Random (MNAR) [2,3]. When missing data occur randomly across all individuals in a study—meaning that each individual has an equal chance of having missing data—this is known as Missing Completely at Random (MCAR) [4]. An example of MCAR would be if data are missing due to random technical errors during data collection. This type of missing data does not introduce bias into the analysis and can often be handled with methods like listwise deletion or mean imputation. MAR occurs when the missingness is related to observed data but not to the missing data themselves [4]. In frailty studies, for example, patients with lower baseline mobility might be less likely to attend follow-up visits. If this non-attendance can be predicted by the observed baseline mobility scores, the data are considered MAR. MNAR is the most challenging scenario, where the missingness is related to the unobserved data themselves [4]. For instance, in a study on frailty, if patients with severe symptoms are more likely to drop out of the study, and this dropout is directly related to their unobserved frailty scores, the data are considered MNAR. Handling MNAR requires complex techniques, such as pattern mixture models or selection models, to properly account for the missingness mechanism. In data preprocessing, handling missing data is a major challenge, especially for machine learning algorithms that require complete datasets to perform effectively. The field has seen a progression from basic to more advanced imputation techniques as the complexity of missing data patterns has increased. Adhikari et al. (2022) [5] provide a thorough survey of imputation strategies specifically designed for IoT datasets, encompassing a range of methods including statistical approaches, machine learning algorithms, deep learning models, and hybrid techniques. Emmanuel et al. (2021) [6] offer an extensive review of machine learning-based imputation methods, detailing their strengths, limitations, and applicability across various data types. In another study, Jegadeeswari et al. (2023) [7] examine different ensemble learning models—such as bagging, boosting, and stacking—emphasizing their effectiveness in converting weak learners into strong predictors for handling missing values. Ma and Chen (2018) [8] focus on recent advancements in Bayesian methods for both ignorable and non-ignorable missing data, discussing models such as the selection model, pattern mixture model, and shared parameter model. Liu et al. (2023) [9] conducted a systematic review assessing the use of deep learning models for data imputation across different types of datasets, highlighting their increasing application in various fields. Furthermore, Sun et al. (2023) [10] carried out a comprehensive numerical study comparing deep learning-based imputation methods, like Generative Adversarial Imputation Networks (GAINs) and Variational Autoencoder (VAE), against more traditional techniques such as multiple imputation by chained equations (MICE) and missForest. In a similar vein, Palli and Devlioti (2023) [11] performed a methodological comparative analysis of 20 machine learning and time-series forecasting algorithms to evaluate the real-time imputation of missing sensor data in maritime applications.

For scenarios where data are Missing Completely at Random (MCAR), simpler methods like deletion or single imputation are often sufficient. Ren et al. (2023) [12] describe MCAR scenarios where missingness is entirely unrelated to any observed or unobserved data, rendering the missing data purely random. In such cases, straightforward approaches like listwise deletion or single imputation (e.g., mean or median imputation) can be effective. However, these methods, while easy to implement, can introduce bias and underestimate variability when the data are not MCAR. To mitigate these limitations, more sophisticated imputation techniques have been developed, utilizing machine learning algorithms to predict the most appropriate values for missing data. For instance, Seu et al. (2022) [13] highlight several cutting-edge imputation methods, including K-Nearest Neighbors imputation (KNNImputer), Bayesian Principal Component Analysis (BPCA), multiple imputation by chained equations (MICE), and the Multiple Imputation with Denoising Autoencoder Neural Network (MIDAS), all of which have shown significant potential in accurately imputing missing values, particularly within medical datasets.

Furthermore, Shadbahr et al. (2023) [14] underscore the importance of imputation quality when developing machine learning classifiers for datasets with missing values. Their research indicates that both the choice of imputation method and the extent of missing data can greatly influence the performance of classifiers. By analyzing a range of datasets with both simulated and real-world missingness, they investigate how different imputation methods—such as mean imputation, missForest, MICE, Generative Adversarial Imputation Networks (GAINs), and the Missing Data Importance-Weighted Autoencoder (MIWAE)—affect not only the accuracy of data reconstruction but also the interpretability of classification models. This highlights the need for robust evaluation criteria to ensure reliable model performance and the accurate assessment of feature importance in predictive modeling.

Effectively handling missing data, particularly when they are not Missing Completely at Random, requires the use of advanced imputation techniques. For data that are Missing at Random (MAR), more sophisticated approaches like multiple imputation are essential to accurately capturing the relationships between observed variables and the patterns of missingness. When practitioners are faced with data that are Missing Not at Random (MNAR), the complexity of the missing data mechanism further requires the application of more advanced statistical models, such as pattern mixture models, to mitigate the potential biases introduced by the missing data. These advanced techniques are crucial for ensuring the robustness and reliability of analyses in the presence of incomplete data.

While significant advancements have been made in applying artificial intelligence and IoT technologies, a critical gap remains in the domain of real-time data imputation for heath monitoring systems. Our previous work [1] primarily focuses on monitoring and analyzing frailty-related parameters in elderly patients through non-intrusive, sensor-based technologies; however, it does not address the challenges of real-time missing data imputation, which is essential for maintaining the reliability and accuracy of data-driven decisions in dynamic environments. The novelty of the current study lies in its targeted exploration of advanced imputation techniques that are specifically designed for real-time applications. By integrating these advanced methods, this study aims to enhance data quality and decision-making processes in critical fields where data are continuously collected and subject to varying patterns of missingness, thus extending the capabilities of systems like the FIDS to ensure more robust, timely, and accurate health assessments and interventions.

A part of the research presented in this paper was conducted as part of the EUREKA research and development project, cINnAMON, titled “A Non-Intrusive Home Surveillance System for Assisting Elderly Individuals at Risk of Frailty”. The cINnAMON project aims to create a non-intrusive and cost-effective home monitoring system designed to support elderly individuals who are vulnerable to frailty.

In this article, we will not only analyze different imputation methodologies in dealing with missing data but also introduce a proprietary data collection system developed in the cINnAMON project designed to monitor frailty in the elderly. This system was developed and fine-tuned in several steps that have already been reported in recent publications. With this custom-built data collection infrastructure, we collected key frailty metrics independently and enriched the platform with simulated missing data scenarios—this allowed us to provide a controlled environment where different imputation methods could be tested and evaluated for their effectiveness.

The cINnAMON project offered significant support to such undertakings by publishing the development of the system in a series of articles: the initial architecture of a non-intrusive IoT system for the detection of frailty [15], prototype results integrating wearables and artificial intelligence for the detection of frailty [16], and the further development of the FIDS dashboard—an intuitive interface enabling real-time health monitoring [1]. Our article contributes to advancing missing data imputation methods in geriatric health monitoring and offers a practical and effective framework for early frailty detection.

Frailty detection systems rely on comprehensive, high-quality data to assess physical and physiological conditions in elderly individuals, enabling timely interventions and improved patient outcomes. Missing data, often resulting from sensor malfunctions or non-compliance, can disrupt the reliability of these systems, leading to misdiagnosis, delayed interventions, or missed opportunities for preventive care. For instance, an inaccurate assessment of physical activity or heart rate variability due to imputed errors might misclassify frailty severity, directly impacting treatment plans or resource allocation.

By improving imputation techniques, this study seeks to enhance the reliability of frailty detection systems. Effective imputation ensures that missing data do not compromise the sensitivity and specificity of these systems, thereby supporting the accurate classification of frailty levels and enabling healthcare providers to make informed decisions. In particular, robust imputation methods facilitate better patient monitoring in real time, reducing false alarms and ensuring actionable insights for clinicians. This connection underscores the critical role of imputation in supporting healthcare outcomes, from individual treatment planning to population-level health management.

## 2. Materials and Methods

This section outlines the dataset, data collection methods, and imputation techniques employed in this research. We gathered physical activity data from wearable devices, focusing on sensors such as accelerometers, gyroscopes, and heart rate monitors to capture activities like running, walking, and climbing stairs. The data were collected at a high frequency and processed to ensure quality, followed by the artificial introduction of missing data to simulate real-world conditions. We implemented various imputation methods—including statistical, machine learning, and deep learning approaches—to address the artificially introduced missing data. Additionally, missing data mechanisms (MCAR, MAR, and MNAR) were simulated to test the robustness of these methods. Finally, we evaluated the imputation methods using normalized metrics (MSE, MAE, and R^2^) to ensure consistent comparisons, providing insights into which techniques best preserved data integrity and maintained the performance of machine learning models for activity classification.

### 2.1. Data Description and Collection Process

**Figure 1:**
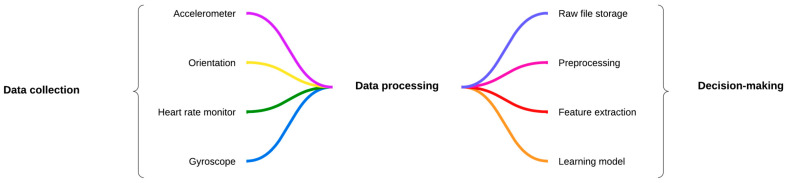
General architecture for data collection.

The dataset utilized in this research is composed of physical activity records obtained from wearable devices, specifically smartwatches equipped with sensors like accelerometers, gyroscopes, and orientation sensors. The dataset includes five primary activities performed by users: running, walking, sitting, standing, and climbing stairs. For each activity, multiple sensor data streams were recorded, as illustrated in Figure 1, including the following:

- Accelerometer data (x, y, z axes): measured the change in the velocity of the user’s movements;
- Gyroscope data (x, y, z axes): captured the angular velocity of the user’s movements;
- Orientation data: included roll, pitch, and yaw, which describe the user’s orientation in space;
- Heart rate monitor (HRM): measured the user’s heart rate in beats per minute (BPM).

The data collection process, as outlined in our previous studies, leveraged an IoT system architecture specifically designed for the monitoring of frailty in elderly individuals, detailed in a series of articles that document its development. Initially, the architecture of a non-intrusive IoT system for frailty detection was established to ensure efficient data capture without interrupting users’ daily activities [15]. This was followed by prototype testing that integrated wearable devices with AI capabilities to enhance the accuracy of frailty detection [16], culminating in the development of the Frailty Insights Detection System (FIDS) dashboard—a user-friendly interface for real-time health monitoring and decision-making [1].

In this setup, activities were continuously recorded with a frequency of 10 readings per second for each sensor; this allowed for a fine-grained view of physical movements. Each activity performed by each participant was carried out for five-minute intervals with breaks in between to avoid sensor fatigue. Data were, therefore, transmitted securely via WebSocket to a cloud server where they were stored for processing and further analysis.

The following steps make up the data processing module, which transforms raw sensor data into actionable insights:

- Raw file storage: the raw data files are stored securely for further processing and historical reference;
- Preprocessing data: this step involves cleaning the raw data to remove any noise or inconsistencies, ensuring that only relevant and high-quality data are used in further steps;
- Feature extraction: specific features are derived from the preprocessed data. For example, mean, variance, and frequency-domain features can be extracted from accelerometer and gyroscope data to represent various aspects of physical movement;
- Learning model: machine learning models are applied to the extracted features to classify physical activities or recognize patterns that are associated with specific actions;
- Decision-making: based on the outputs from the learning model, a decision is made regarding the detected activity (e.g., classifying the current movement as running, walking, sitting, standing, and climbing stairs). This decision-making process is crucial for real-time activity recognition.

After processing and decision-making, the system classifies the user’s activities, and results can then be used for further analysis and monitoring, or even as feedback to healthcare providers or researchers. These features are labeled with the type of activity performed. The data include timestamps and continuous sensor recordings, with activity labels annotated in real time. The dataset provides a rich basis for evaluating imputation techniques in a controlled environment where missing data are introduced artificially.

### 2.2. Artificial Introduction of Missing Data and Imputation Methods

In the next phase of the research, artificially missing data are introduced into the existing dataset to simulate real-world scenarios where data might be incomplete due to various factors such as sensor malfunctions, user non-compliance, or environmental conditions. Missing data are randomly removed (10% of data) to test the effectiveness of different imputation methods.

The 10% missingness rate was chosen to reflect the typical data loss observed in wearable health monitoring systems, often caused by device malfunctions or user non-compliance. For example, a study analyzing heart rate sensor data from consumer-grade wearables reported a missingness rate of approximately 10.4% during physical activity [17]. This rate was applied in a controlled, one-time process to maintain computational feasibility while ensuring real-world relevance. Future work will expand this analysis by considering multiple simulations and varying levels of missingness (e.g., 20%, 30%) to evaluate the scalability and robustness of the proposed methods under different data loss scenarios.

The following imputation methods were applied to handle the artificially introduced missing data:

- Expectation–Maximization (EM): a statistical method that iteratively estimates missing data by maximizing the likelihood of observed values.
- Matrix Completion Methods: techniques that estimate missing values by leveraging low-rank approximations of the data matrix.
- Bayesian Networks: probabilistic models that infer missing values based on the relationships between observed data points.
- K-Nearest Neighbors (KNN): a method that imputes missing values by finding the most similar data points and averaging their values.
- Support Vector Machine (SVM): a machine learning approach that estimates missing data by finding optimal decision boundaries within the observed data.
- GAIN (Generative Adversarial Imputation Network): a technique that uses adversarial learning to model the distribution of missing data.
- VAE (Variational Autoencoder): a neural network-based method that learns a latent representation of the data for imputation.
- GRU-D (Gated Recurrent Unit with Decay Mechanism): a recurrent neural network method tailored for time-series data imputation, adjusting for data decay over time.

**Figure 2:**
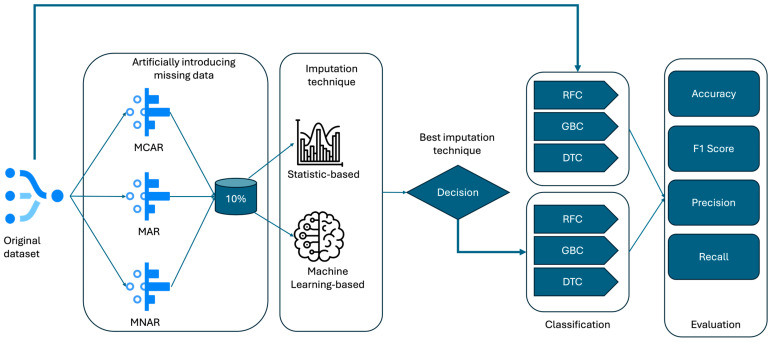
Research methodology.

After the missing data were imputed using these methods, three machine learning model types (Random Forest, Gradient Boosting, and Decision Tree Classifiers) were trained (for better readability, they will be referred to as post-imp models) and compared with the same models (different model instances for each model type, referred to as baseline models) trained with the initial dataset, as we see in the Figure 2. The performance of the post-imp models was compared with the results from the original dataset (baseline models) without missing data. The goal of this was to assess the effectiveness of each imputation method by evaluating and comparing relevant performance metrics—accuracy, precision, recall, and F1 scores—from the models applied to both the imputed and original datasets. This comparison allowed for the determination of which imputation method best preserved the dataset’s integrity and enabled the models to maintain or improve their performance metrics.

### 2.3. Artificially Introducing Missing Data

To evaluate the robustness and performance of various imputation techniques, three types of missing data mechanisms were simulated: Missing Completely at Random (MCAR), Missing at Random (MAR), and Missing Not at Random (MNAR). The purpose of this approach was to reflect the variety of ways that data may be lost in real-world sensor-based systems and to test the efficacy of different imputation techniques under these conditions.

#### 2.3.1. MCAR—Missing Completely at Random

In the MCAR scenario, data were removed randomly across the dataset. This simulated situations where data loss occurred independently of any other variables, such as due to random device malfunctions or the accidental loss of sensor readings. A total of 10% of the sensor-recorded data (accelerometer and gyroscope readings) were randomly set to missing values. This scenario assumes that there is no systematic pattern in the missing data, meaning that the probability of data being missing is equal for all observations.

#### 2.3.2. MAR—Missing at Random

In the MAR scenario, missing data were introduced based on the values of another observed variable. Specifically, missing data were introduced into the Accelerometerx, Accelerometery, and Accelerometerz columns, with the missingness depending on the heart rate data (hrm column). This scenario reflects a real-world situation where data from an accelerometer may be more likely to be lost or unavailable when a user’s heart rate exceeds a certain threshold, such as during periods of intense physical activity. In our approach, we sorted the dataset by hrm values (heart rate), and missing data were introduced into the accelerometer readings for the top 10% of rows where the highest heart rates were recorded. This approach simulated a real-world scenario where higher physical exertion could cause increased data loss or sensor malfunction.

#### 2.3.3. MNAR—Missing Not at Random

In the MNAR scenario, missing data were introduced such that the missingness depended directly on the values of the missing data themselves. For instance, in the case of accelerometer data, we introduced missing values in the Accelerometerx, Accelerometery, and Accelerometerz columns based on the recorded values of these columns themselves. This simulated a situation where the likelihood of missing data increased with specific ranges of sensor readings—such as during high or low accelerations, where the sensor may fail to record the data accurately. For this, we sorted the dataset based on the values of the accelerometer columns and removed data for the top or bottom ranges of the distribution, reflecting scenarios where sensor readings are more likely to be lost during extreme accelerations or movements.

### 2.4. Imputation Technique Analysis

#### 2.4.1. Statistic-Based

The Expectation–Maximization (EM) algorithm has been extensively applied in handling missing data, especially under the Missing Completely at Random (MCAR) and Missing at Random (MAR) conditions. EM operates by iteratively estimating the missing values while simultaneously maximizing the likelihood function of the observed data. According to Nguyen (2021) [18], EM can effectively handle missing data by treating them as hidden data and establishing an implicit relationship between the observed data and the missing values through joint distribution or mapping functions. Nguyen emphasizes the theoretical basis of EM, providing detailed mathematical proofs to support its use for imputing missing values, particularly in datasets following multinormal or multinomial distributions. Similarly, Chen et al. (2019) [19] highlight the application of EM in Single-Case Experimental Design (SCED) studies, where missing data are commonly encountered due to multiple measures of target behaviors. Their study extends prior work by systematically examining EM’s performance under various conditions, such as different missingness rates and autocorrelations.

Matrix completion methods have proven to be effective in addressing missing data, particularly under MCAR and MAR conditions. In a study by Arciniegas-Alarcón et al. (2023) [20], regularized singular value decomposition (SVD) was used to impute missing values in agricultural datasets, showing significant improvements in datasets with various levels of missing data. Although the study did not explicitly focus on MCAR, MAR, or MNAR conditions, the structured relationships between variables in the data suggest their strength under MCAR and MAR conditions. Similarly, Huang et al. (2022) [21] developed the single-cell Gene Network Guided Imputation (scGNGI) algorithm, a matrix completion method specifically designed for single-cell RNA sequencing (scRNA-seq) data, which addresses missing values likely caused by MAR mechanisms (where missingness is related to gene expression variability). Their results demonstrated that scGNGI preserved heterogeneity in the data and outperformed other imputation methods. Zhang et al. (2024) [22] applied matrix completion in handling missing sensor data in chemical processes, where missingness is often random or related to observable factors, making matrix completion effective in MCAR and MAR conditions.

Bayesian networks are flexible tools for exploring complex causal relationships between variables. The application of Bayesian networks to data with missing values is enhanced when imputation techniques are integrated. For example, Ke et al. (2021) [23] explored Bayesian network structure learning using the Structural Expectation–Maximization (SEM) algorithm, comparing it with multiple imputation by chained equations (MICE). Their findings indicated that SEM outperforms MICE across various missing data mechanisms (MCAR, MAR, and MNAR) due to its use of additional network structure information during imputation. Additionally, Howey et al. (2021) [24] highlighted the potential of Bayesian networks for biological data by incorporating an imputation method that selects nearest neighbors to replace missing data, improving the network’s recall and precision. Their approach is particularly useful in complex biological datasets, offering marked improvements in detecting causal relationships.

In both cases, the Bayesian network approach, particularly when combined with advanced imputation techniques like SEM and nearest neighbor-based methods, provides a robust framework for handling missing data, whether they fall under MAR, MCAR, or MNAR. The flexibility of Bayesian networks in accounting for conditional dependencies enhances the accuracy of causal inference even when missing data are present.

#### 2.4.2. Machine Learning-Based

K-Nearest Neighbors (KNN) imputation is a widely used method for handling missing data by utilizing the similarity between data points. Rizvi et al. (2023) [25] demonstrate that KNN is particularly effective when data are Missing at Random (MAR), as it can predict missing values by identifying similar data points based on observed variables, making it more accurate than when data are completely random. While KNN can still be applied to Missing Completely at Random (MCAR) data, its performance is less reliable, as the missingness is independent of observed and unobserved values. Petrazzini et al. (2021) [26] further discuss that KNN’s accuracy declines when dealing with Missing Not at Random (MNAR) data, where missingness is related to unobserved variables, but reasonable results can still be achieved depending on assumptions about the underlying structure. Similarly, Pan et al. (2015) [27] highlight that KNN is most effective for MAR data, as it leverages the relationships between observed data points to provide accurate imputations. However, for MCAR data, KNN’s performance decreases as the missingness lacks any correlation with the data, and for MNAR data, KNN struggles unless specific assumptions about the missingness are made. Overall, KNN is best suited for MAR scenarios, with more limited use in MCAR and MNAR data without additional modifications.

Support Vector Machines (SVMs) have been proven to be a highly effective method for handling missing data in various machine learning applications. Li et al. (2024) [28] demonstrate that SVM, when used for predictive modeling in cohort studies, performs well in imputing missing data, particularly when dealing with Missing at Random (MAR) data. Their study, which evaluated several imputation techniques, found that SVM models showed high performance in predictive accuracy, especially for cardiovascular disease risk prediction models. Palanivinayagam and Damaševičius (2023) [29] also highlight the success of SVM in imputing missing data in classification tasks. Their research emphasizes that SVM-based imputation helps improve classification accuracy by substituting missing values based on the relationships between observed data points. This is particularly useful when missingness is related to other observed variables.

Pelckmans et al. (2005) [30] further extend the application of SVM by introducing robust SVM-based methods for imputation in Missing Not at Random (MNAR) settings. Although they are more challenging, their work demonstrates that SVM models can still offer reasonable performance by learning from the structure of available data, especially when assumptions about the missing data mechanism are incorporated. In summary, SVM imputation is most effective in MAR scenarios, but it can also handle MNAR data under specific conditions, making it a versatile tool for addressing missing data challenges.

Deep learning methods like GAIN (Generative Adversarial Imputation Network), VAE (Variational Autoencoder), and GRU-D (Gated Recurrent Unit with Decay Mechanism) offer distinct advantages for handling missing data, depending on whether the missingness is MCAR, MAR, or MNAR. GAIN, explored by Dong et al. (2021) [31], excels in MAR settings where missingness is related to observed variables. GAIN uses an adversarial process to generate realistic imputations, outperforming traditional methods like MICE and missForest, particularly in large clinical datasets with mixed data types and high missingness rates. VAE, as discussed by McCoy et al. (2018) [32], performs robustly in MCAR scenarios by learning latent representations that can model the underlying structure of the data. VAE is also effective for MAR data but struggles with MNAR data, where missingness depends on unobserved factors.

GRU-D, introduced by Che et al. (2018) [33], is most effective in MNAR scenarios, where the missingness pattern is informative. By incorporating masking and time intervals directly into the recurrent neural network, GRU-D leverages the missing patterns to improve predictions in multivariate time series, such as those seen in clinical datasets. It also performs well in MAR situations, but its effectiveness in MCAR situations is more limited, as the model is designed to exploit informative missingness.

Based on the analysis in Table 1, it is clear that no single imputation technique is universally suited for all types of missing data (MCAR, MAR, and MNAR). Each method has its strengths and weaknesses, depending on the missing data mechanism. Given that each technique has unique strengths, combining different methods could indeed be a powerful approach to improving the overall imputation process. By strategically combining these techniques, we can tackle datasets with mixed missing data types and achieve more robust imputation results. This hybrid approach would allow us to maximize the benefits of each method and compensate for their individual weaknesses.

### 2.5. Evaluation Metrics

According to a study by Chicco D et al. (2021) [34], metrics like Mean Squared Error (MSE), Mean Absolute Error (MAE), and R^2^ are very important in the evaluation of the quality of imputed data, especially in the medical field, where precision is key for decision-making.

We evaluated all the imputation methods using the following three metrics, which offered an overview of imputation quality:

- MSE: This penalizes large errors more than smaller ones. This is critical in medical cases because outliers or large deviations in data—like very high or very low heart rates or movements—may result in wrong clinical decisions being made;
- MAE: this provides an intuitive understanding of the average error magnitude and will be very useful in understanding the general accuracy level of imputation across all observations;
- R^2^ (coefficient of determination): this describes how well the imputed data fit the original data. It is especially useful in looking at how well an imputation method can keep underlying patterns in a dataset, which is very important to assuring that the imputed data do not lose their meaning within patient monitoring.

In order to compare the different imputation methods, we used normalized versions of MSE, MAE, and R^2^. Among those, normalization enables consideration of the differences in scale present in the original data and sets a common framework under which one can compare performances on different datasets or with different imputation techniques. In the context of our study, normalizing these metrics ensured that imputation methods were compared based on relative effectiveness rather than being skewed by the variance in or distribution of the sensor data. This step is especially important in processing diverse data from different sensors; for example, accelerometer readings have a different scale and range compared to heart rate data.

This process scales the metrics to a range between 0 and 1, enabling a consistent evaluation of performance across methods that may have differing scales or units.

The *Min-Max* normalization formula applied is as follows:

$$\mathrm{Normalized}\;\mathrm{value}=\frac{\mathrm{Value}-\mathrm{Min}}{\mathrm{Max}-\mathrm{Min}}$$

where

- Value is the original MSE or MAE for a specific imputation method;
- Min and Max represent the smallest and largest MSE or MAE values across all methods, respectively.
- This normalization process ensures the following:
- The best-performing method for a given metric has a normalized value of 0 (smallest error);
- The weakest-performing method has a normalized value of 1 (largest error);
- All other methods are proportionally scaled between these extremes.

By applying normalization, we harmonized the evaluation metrics, particularly since $R^{2}$ inherently ranges between 0 and 1, while MSE and MAE can vary significantly in magnitude. This approach facilitated direct comparison and enhanced the interpretability of the results, as visualized in the bar charts. The normalized metrics allowed for a clear and unbiased assessment of each imputation method’s relative performance, highlighting trends and differences effectively.

To ensure robust and reproducible results, we performed hyperparameter tuning for the KNN and SVM imputation methods, tailoring each to the characteristics of the dataset. For KNN, the tuning process focused on optimizing the number of neighbors (n_neighbors) and the distance metric (metric). We explored candidate values for n_neighbors in the range of [3,5,10] and evaluated different distance metrics, including ‘euclidean’ and ‘manhattan’. For SVM, key hyperparameters such as the regularization parameter (C), epsilon (epsilon), and kernel type (kernel) were optimized. The ranges for these parameters were chosen to balance model complexity and performance: C values of [0.1, 1, 10], epsilon values of [0.01, 0.1, 0.5], and kernel types including ‘rbf’ and ‘linear’. A grid search with 5-fold cross-validation was employed for both methods, using non-missing data as the validation set and selecting the best configurations. This systematic approach ensured that the imputation process was both effective and reproducible across different missing data patterns.

For both KNN and SVM, cross-validation was conducted independently for each missing data mechanism (MCAR, MAR, and MNAR) to ensure that the chosen hyperparameters were robust across diverse missingness patterns. All experiments were implemented using Python’s (3.12.5) Scikit-learn library, with hyperparameter optimization performed through the GridSearchCV module.

## 3. Results

In our work, we introduced artificially missing data to our dataset, collected through smart bracelets used in a medical context, with the purpose of evaluating various imputation techniques used to estimate physical activity and monitor vital signs in elderly individuals. Wearable devices in healthcare can have missing accelerometer and heart rate values due to device malfunction, user non-compliance, and environmental factors. These incomplete data considerably affect the reliability of any downstream analysis or predictive models focused on monitoring patient health and frailty. We introduced missing data under three mechanisms to assess the robustness of different imputation methods: MCAR, MAR, and MNAR. These mechanisms were described in the article as conditions where the missing data occur in an absolutely unpredictable way, are related to some observed variables, or are linked to the missing data themselves. To better approximate these realistic scenarios that may arise during the use of smart bracelets in a healthcare environment, we simulated these missing data mechanisms.

### 3.1. Imputation Method Performance Across Missing Data Mechanisms

#### 3.1.1. MAR (Missing at Random)

**Figure 3:**
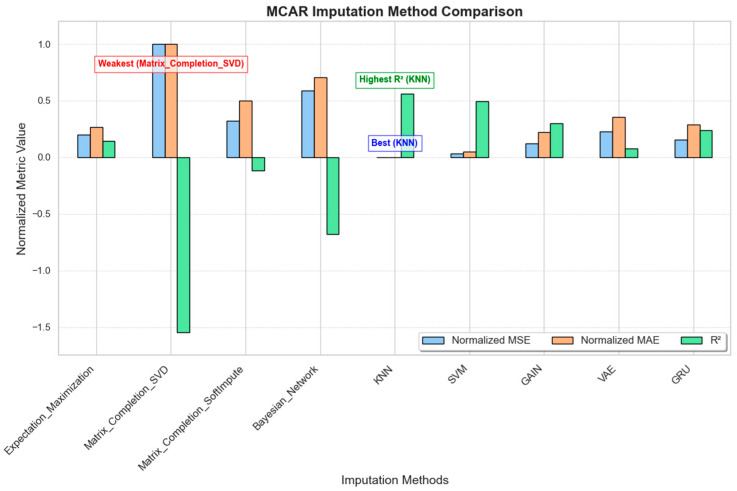
MAR imputation method comparison.

In the MAR scenario, missingness depended on observed variables, simulating conditions where, for example, sensor data may be more likely to be missing at high heart rates. Figure 3 illustrates the normalized MSE, MAE, and R^2^ values for each imputation method under the MAR scenario.

- KNN and SVM show the best performance, with the lowest MSE and MAE values and the highest R^2^ scores. These results indicate that KNN and SVM are the most effective methods for MAR data, providing accurate imputations and closely fitting the original data structure;
- GRU, GAIN, and VAE also perform reasonably well, with moderate error values and positive R^2^ scores, though they are slightly less effective than KNN and SVM;
- Matrix completion methods (SVD and SoftImpute) show poor performance with high error values and negative R^2^ scores, suggesting that they are unsuitable for MAR scenarios;
- Expectation–Maximization (EM) and Bayesian networks perform moderately, with error values higher than KNN and SVM and with slightly negative or low positive R^2^ scores, indicating a limited ability to capture the data’s structure accurately.

These results highlight that KNN and SVM provide the best balance between low error rates and high data fidelity in MAR scenarios.

#### 3.1.2. MCAR (Missing Completely at Random)

**Figure 4:**
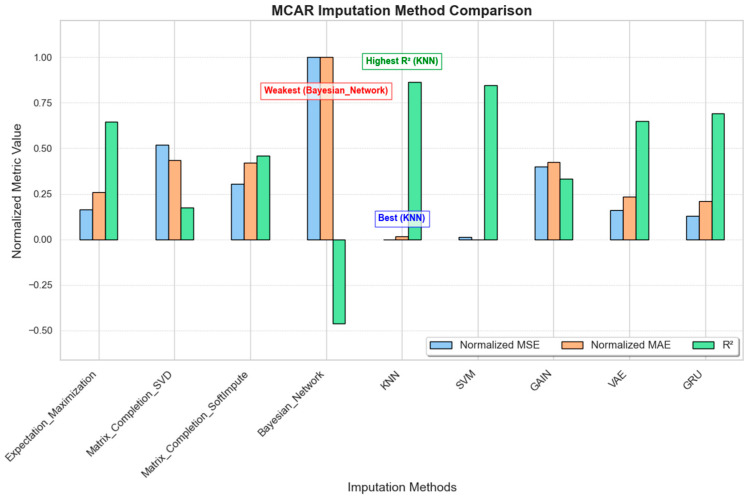
MCAR imputation method comparison.

The MCAR scenario introduced missing data at random across the dataset, simulating potential random device malfunctions. Figure 4 shows the performance of various imputation methods under MCAR conditions.

- KNN and SVM demonstrate the best performance, with the lowest normalized MSE and MAE values and the highest R^2^ scores, indicating their effectiveness in handling random missingness;
- VAE, Expectation–Maximization, and GRU also perform relatively well, achieving low error rates and positive R^2^ scores, suggesting that they provide reasonable accuracy in imputing random missing values;
- GAIN, SVD, and SoftImpute show moderate performance, though with slightly higher error values;
- The Bayesian network shows particularly poor performance, with high error values and a significantly negative R^2^ score, suggesting that it is not well suited for MCAR scenarios.

In summary, KNN and SVM are the most suitable methods for MCAR data, with VAE and GRU offering acceptable accuracy as secondary options.

#### 3.1.3. MNAR (Missing Not at Random)

**Figure 5:**
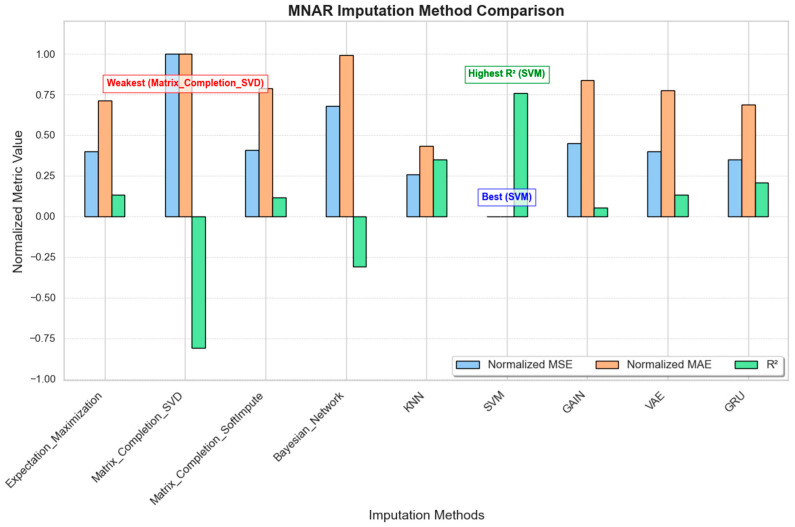
MNAR imputation method comparison.

In the MNAR scenario, missingness depended on the unobserved data themselves, simulating situations where certain sensor readings (e.g., high accelerations) are more likely to be missing. Figure 5 illustrates the performance of each imputation method under MNAR conditions.

- SVM performs the best among all methods, showing the lowest error rates (MSE and MAE) and a high R^2^ score, which suggests that it handles MNAR data very effectively;
- KNN also performs well, with low error rates and a positive R^2^ score, meaning that it can manage missing data that depend on unobserved factors reasonably well;
- VAE, GRU, and Expectation–Maximization show average results. They work for MNAR data to an extent, but they do not match the effectiveness of SVM and KNN.
- GAIN and SoftImpute struggle in MNAR conditions, with higher error rates indicating that they are not well suited to this type of data.
- Matrix completion (SVD) and the Bayesian network perform poorly, with high error values and negative R^2^ scores, making them the least suitable options for MNAR data.

In summary, SVM is the most suitable choice for handling MNAR data, followed by KNN, while VAE, GRU, and Expectation–Maximization provide moderate performance.

An Analysis of Variance (ANOVA) was conducted to evaluate the effectiveness of imputation methods under three missing data mechanisms: Missing Completely at Random (MCAR), Missing at Random (MAR), and Missing Not at Random (MNAR).

Table 2 summarizes the F-statistic and *p*-values for three key performance metrics: Mean Squared Error (MSE), Mean Absolute Error (MAE), and the coefficient of determination ($R^{2}$). The F-statistic quantifies the ratio of between-group variability to within-group variability, with higher values indicating greater differences between methods. The *p*-values (all <0.001) indicate that the observed differences between imputation methods are statistically significant for all metrics and missing data mechanisms.

These results validate the finding that the imputation methods differ significantly across all metrics and missing data mechanisms. Detailed pairwise comparisons for each metric and mechanism are provided in the Supplementary Materials.

### 3.2. Machine Learning Model Performance Comparison

In healthcare applications, particularly those involving continuous monitoring through wearable devices, missing data are frequent and critical issues that can undermine the reliability of predictive models and overall data integrity. Device malfunctions, patient non-compliance, and environmental interference can all contribute to data gaps, which must be addressed to ensure that machine learning models can make accurate and actionable predictions.

In this study, we examined a range of imputation techniques to determine their suitability for handling missing data under three different scenarios: Missing at Random (MAR), Missing Completely at Random (MCAR), and Missing Not at Random (MNAR). Each of these scenarios represents a distinct type of missingness commonly encountered in medical data. After evaluating the performance of multiple imputation methods in the previous section, including Expectation–Maximization (EM), matrix completion (SVD and SoftImpute), Bayesian networks, K-Nearest Neighbors (KNN), Support Vector Machines (SVMs), Generative Adversarial Imputation Network (GAIN), Variational Autoencoder (VAE), and GRU-D, we concluded that KNN and SVM consistently provided the most reliable imputation across the different missing data types.

KNN and SVM proved to be particularly effective for handling missing data in our study. KNN is useful because it fills in gaps by comparing similar data points, making it especially suitable for MAR and MCAR scenarios. SVM, meanwhile, was able to manage MAR and MCAR cases well and showed an added advantage in MNAR scenarios, where missingness depends on factors that we cannot directly observe. This flexibility made SVM and KNN stand out from the other methods we tested, so we chose to focus on further evaluation of these two techniques.

In this section, we present the results of training three machine learning models—the Decision Tree Classifier, Gradient Boosting Classifier, and Random Forest Classifier—on both the original complete dataset and the datasets imputed using KNN and SVM for each missing data scenario (MAR, MCAR, and MNAR). By comparing model performance across these different datasets, we aimed to identify the imputation method that best preserved the dataset’s integrity and supported robust model predictions, thereby providing insight into the most effective imputation strategies for real-world healthcare data.

Initially, we referenced baseline performance metrics from a previous study [16], where each model was trained on a complete dataset with no missing values. These metrics provided a reference for evaluating how well each imputation method maintained model accuracy, precision, recall, and F1 scores after addressing missing data. The baseline results are presented in Table 3.

In our previous research detailed in Ciubotaru et al. (2023) [16], we conducted a thorough evaluation of multiple machine learning models to identify the most effective classifiers for accurately detecting and categorizing different types of physical activities. Through this evaluation, we found that the Decision Tree Classifier (DTC), Random Forest Classifier (RFC), and Gradient Boosting Classifier (GBC) models demonstrated superior reliability and stability in their performance metrics.

The selection of these models was based on a detailed comparison against other widely used algorithms, including Logistic Regression (LR), K-Nearest Neighbors (KNN), the Support Vector Classifier (SVC), and Gaussian Naive Bayes (GNB). These alternative models are popular in machine learning applications and often perform well across various classification tasks. However, when applied to our specific context of activity recognition, these models did not exhibit the same level of consistent performance as did the DTC, RFC, and GBC. Each of these top-performing classifiers showed remarkable accuracy, precision, recall, and F1 scores, which are essential for high-quality activity detection.

The scores presented in Table 3 reflect the baseline performance of each model on the complete dataset, representing the optimal accuracy, precision, recall, and F1 scores without any imputation adjustments. These values serve as a benchmark to assess how effectively imputed data can support predictive performance comparable to that of the original complete dataset.

After introducing missing data into the dataset, we applied KNN imputation and re-evaluated each model across MAR, MCAR, and MNAR scenarios. Table 4 summarizes the performance metrics of the models on the KNN-imputed data.

The results in Table 4 demonstrate that KNN imputation preserved a high level of predictive performance across all models and scenarios, with the MAR scenario showing the most consistent improvement. This indicates that KNN is particularly effective when missing data are related to other observed variables, as it can approximate values that closely align with the original dataset’s structure.

Similarly, we evaluated the models on datasets imputed with SVM under each missing data mechanism, with the results presented in Table 5.

The SVM-imputed data also maintained robust model performance, particularly in MAR and MCAR scenarios. SVM provided reliable imputation across missing data types, although slightly lower metrics were observed under MNAR conditions, suggesting that SVM may be more suited to situations where missingness is associated with observed variables rather than unobserved factors.

Both imputation techniques demonstrated strong performance across all missing data scenarios, with KNN and SVM achieving the highest metrics for MAR and MCAR data. This reinforces their potential utility in healthcare settings, where accurate data recovery is essential. The Decision Tree Classifier showed significant improvement in all metrics with both KNN and SVM imputations, particularly under MAR conditions. Gradient Boosting and Random Forest Classifiers also exhibited substantial gains in recall and F1 scores, indicating their robustness in and adaptability to imputed datasets.

KNN performed exceptionally well for MAR data, while SVM showed slightly better alignment with MCAR data. The moderate performance under MNAR conditions for both methods suggests that further work may be needed to develop hybrid techniques that more effectively handle dependent missingness patterns.

## 4. Discussion and Conclusions

In healthcare applications, especially those involving continuous monitoring through wearable devices, missing data pose a critical challenge that can significantly impact the accuracy and reliability of predictive models. This issue is particularly relevant in geriatric health monitoring, where wearable devices, like smart bracelets, are frequently used to assess physical activity and vital signs in elderly individuals. Missing data in this context can arise due to device malfunctions, patient non-compliance, or environmental factors, leading to gaps that must be addressed to ensure robust and actionable insights.

This study introduced artificially missing data under three distinct mechanisms—MCAR, MAR, and MNAR—to assess the robustness and effectiveness of various imputation techniques in managing data gaps typical of real-world healthcare scenarios. Following a comprehensive evaluation of multiple imputation methods, including Expectation–Maximization, matrix completion, Bayesian networks, KNN, SVM, GAIN, VAE, and GRU-D, we found that KNN and SVM consistently outperformed the others across the different types of missing data, making them the most reliable choices for this study.

In our study, we focused specifically on the practical challenges of using wearable devices to monitor the health of elderly patients, particularly in frailty tracking—an area where missing data has direct implications for patient care. Unlike in other settings, gaps in this type of data can significantly impact the reliability of health assessments. Foundational frameworks by Graham [2] and Rubin [3] introduced the concepts of MCAR, MAR, and MNAR to classify types of missing data, which remain widely used. Building on these concepts, our study applied them within the context of real-time health data from wearable sensors, a scenario where missing data patterns pose unique, immediate challenges to effective healthcare.

While studies by Adhikari et al. [5] and Liu et al. [9] address data imputation in broader IoT and healthcare settings, our research specifically examined sensor data from wearable devices used by older adults. This focus introduces distinct challenges, as elderly users may wear these devices inconsistently, and technical malfunctions are frequent, leading to unique data gaps that are not often accounted for in more generalized studies. Our findings indicate that KNN and SVM methods are particularly effective in managing these missing data types, especially in MAR and MCAR scenarios, which commonly arise due to factors like device non-compliance or random sensor failures.

While KNN and SVM demonstrate robust performance across all missing data mechanisms, their effectiveness in MNAR scenarios has limitations. Specifically, KNN relies on observed data relationships, which may not fully capture unobserved dependencies, and SVM’s decision boundaries can lead to biased imputations when missingness is dependent on unobserved factors.

To address these limitations, future work could explore hybrid imputation methods that combine the strengths of multiple techniques, such as integrating SVM or KNN with models like GRU-D, which excels in leveraging temporal patterns and unobserved data dependencies. Additionally, real-time implementation challenges, including computational efficiency and seamless integration with healthcare systems, require further investigation. Evaluating these methods based on larger and more diverse datasets will be critical to improving their generalizability and applicability in real-world healthcare scenarios.

Additionally, unlike studies by Emmanuel et al. [6] and Sun et al. [10], which evaluate KNN and SVM for general predictive modeling, we tested these methods specifically in a healthcare setting where missing data often correlates with key health metrics like heart rate or activity level. This correlation adds complexity, as it requires not only filling in data gaps but also maintaining the integrity of health trends over time. Our results show that KNN and SVM can achieve this, making them especially suited to applications where maintaining data quality is essential for meaningful health monitoring. These findings could be particularly useful in designing health systems that need to handle missing data without compromising patient insights.

In this study, we focused on foundational technical metrics, such as normalized MSE, MAE, and R2R2, to evaluate the performance of imputation methods. However, we acknowledge the importance of domain-specific metrics to bridge the gap between technical results and practical healthcare applications. Future work will explore the integration of metrics that assess the clinical utility and real-world impact of imputed data. For instance, we plan to evaluate how imputation quality influences healthcare decisions by leveraging simulated clinical datasets and expert feedback. Additionally, clinician-driven assessments will be conducted to validate the reliability of imputation techniques for specific use cases, such as remote frailty monitoring and fall risk prediction. This expansion will enable a deeper understanding of how imputation methods can support critical healthcare outcomes, such as by improving patient safety and enhancing decision-making accuracy.

In summary, our study builds on the foundation laid by previous research by focusing on the particular requirements of frailty monitoring and demonstrating the effectiveness of KNN and SVM under realistic conditions in geriatric care. This targeted approach adds a valuable dimension to the field, supporting the use of these imputation methods in scenarios where patient outcomes heavily rely on data completeness and accuracy.

In conclusion, this study offers a comprehensive evaluation of imputation techniques specific to geriatric health monitoring, demonstrating that selecting an imputation method based on the missing data mechanism is essential for ensuring data integrity. The results provide strong evidence for continued research into advanced and hybrid imputation strategies, which will be crucial for translating these findings into reliable, production-ready systems for sensitive healthcare applications like cINnAMON.

## Figures and Tables

**Table 1:** Comparative suitability of imputation techniques across different missing data mechanisms (MCAR, MAR, and MNAR).

Imputation Technique	Best Suited for MCAR	Best Suited for MAR	Best Suited for MNAR
Expectation–Maximization (EM)	Effective	Effective	Less effective
Matrix Completion (e.g., SVD, scGNGI)	Effective	Effective	Less effective
Bayesian Networks (SEM)	Effective	Effective	Effective when using SEM or advanced imputation techniques
K-Nearest Neighbors (KNN)	Less reliable	Effective	Struggles unless assumptions are made
Support Vector Machines (SVMs)	Moderate	Effective	Effective under specific conditions (with assumptions)
GAIN (Generative Adversarial Imputation Network)	Moderate	Highly effective	Struggles
VAE (Variational Autoencoder)	Highly effective	Effective	Struggles
GRU-D (Gated Recurrent Unit with Decay)	Less effective	Effective	Highly effective, exploits informative missingness

**Table 2:** Summary of ANOVA results for imputation method performance across missing data mechanisms.

Mechanism	Metric	F-Statistic	*p*-Value
MCAR	MSE	236.78	<0.001
	MAE	53.69	<0.001
	$R^{2}$	178.45	<0.001
MAR	MSE	157.68	<0.001
	MAE	123.88	<0.001
	$R^{2}$	195.57	<0.001
MNAR	MSE	346.05	<0.001
	MAE	625.71	<0.001
	$R^{2}$	236.78	<0.001

**Table 3:** Baseline performance metrics of machine learning classifiers on complete dataset.

Algorithm	Accuracy	Precision	Recall	F1 Score
Decision Tree Classifier	0.93318223	0.932582389	0.93318223	0.93248249
Gradient Boosting Classifier	0.962103354	0.961588192	0.962103354	0.961205918
Random Forest Classifier	0.962103354	0.961879967	0.962103354	0.960698886

**Table 4:** Performance metrics of machine learning classifiers on KNN-imputed dataset.

Type	Algorithm	Accuracy	Precision	Recall	F1 Score
MAR	Decision Tree Classifier	0.957087126	0.958283156	0.957087126	0.957502299
	Gradient Boosting Classifier	0.973862159	0.972069266	0.973862159	0.97182681
	Random Forest Classifier	0.975552666	0.973989576	0.975552666	0.973228639
MCAR	Decision Tree Classifier	0.954746424	0.954049264	0.954746424	0.954053196
	Gradient Boosting Classifier	0.970091027	0.967924456	0.970091027	0.967770363
	Random Forest Classifier	0.971131339	0.969205463	0.971131339	0.968344367
MNAR	Decision Tree Classifier	0.932728921	0.931907571	0.932728921	0.931919506
	Gradient Boosting Classifier	0.962556664	0.962053907	0.962556664	0.961698273
	Random Forest Classifier	0.960652765	0.960375385	0.960652765	0.959273856

**Table 5:** Performance metrics of machine learning classifiers on SVM-imputed dataset.

Type	Algorithm	Accuracy	Precision	Recall	F1 Score
MAR	Decision Tree Classifier	0.953185956	0.953460656	0.953185956	0.952842459
	Gradient Boosting Classifier	0.973602081	0.972103834	0.973602081	0.971758387
	Random Forest Classifier	0.972041612	0.970217565	0.972041612	0.969446292
MCAR	Decision Tree Classifier	0.94863459	0.947808078	0.94863459	0.947837256
	Gradient Boosting Classifier	0.969180754	0.967204602	0.969180754	0.966702258
	Random Forest Classifier	0.968335501	0.96733598	0.968335501	0.964875649
MNAR	Decision Tree Classifier	0.95006502	0.949793083	0.95006502	0.949606313
	Gradient Boosting Classifier	0.971391417	0.969575556	0.971391417	0.969308699
	Random Forest Classifier	0.969310793	0.967989494	0.969310793	0.966321118

## Data Availability

A collection of data with all datasets used in this research can be found at https://doi.org/10.6084/m9.figshare.c.7538409.v1. This collection includes the following: original data with missing values—raw datasets with artificially introduced missing values under MCAR, MAR, and MNAR conditions; imputed data (KNN and SVM)—datasets imputed using KNN and SVM methods, allowing for comparison of imputation techniques; machine learning models—after imputation, three classifiers (Random Forest, Gradient Boosting, and Decision Tree) were trained on both imputed and original data, referred to as post-imputed and pre-imputed models, respectively.

## Supplementary Materials

The following supporting information can be downloaded at https://www.mdpi.com/article/10.3390/s25030614/s1, ANOVA summary tables: full ANOVA results for MSE, MAE, and $R^{2}$ under MAR, MCAR, and MNAR scenarios. Pairwise comparisons: tables providing detailed pairwise comparisons of methods, highlighting significant differences for each metric and mechanism.
